# Thermal stress and mutation accumulation increase heat shock protein expression in *Daphnia*

**DOI:** 10.1007/s10682-022-10209-1

**Published:** 2022-09-06

**Authors:** Henry Scheffer, Jeremy E. Coate, Eddie K. H. Ho, Sarah Schaack

**Affiliations:** Department of Biology, Reed College, 3203 SE Woodstock Blvd, Portland, OR 97202 USA

**Keywords:** Stress response, Global climate change, HSP60, HSP90, Waterfleas

## Abstract

Understanding the short- and long-term consequences of climate change is a major challenge in biology. For aquatic organisms, temperature changes and drought can lead to thermal stress and habitat loss, both of which can ultimately lead to higher mutation rates. Here, we examine the effect of high temperature and mutation accumulation on gene expression at two loci from the heat shock protein (HSP) gene family, HSP60 and HSP90. HSPs have been posited to serve as ‘mutational capacitors’ given their role as molecular chaperones involved in protein folding and degradation, thus buffering against a wide range of cellular stress and destabilization. We assayed changes in HSP expression across 5 genotypes of *Daphnia magna*, a sentinel species in ecology and environmental biology, with and without acute exposure to thermal stress and accumulated mutations. Across genotypes, HSP expression increased ~ 6× in response to heat and ~ 4× with mutation accumulation, individually. Both factors simultaneously (lineages with high mutation loads exposed to high heat) increased gene expression ~ 23×—much more than that predicted by an additive model. Our results corroborate suggestions that HSPs can buffer against not only the effects of heat, but also mutations—a combination of factors both likely to increase in a warming world.

**Supplementary Information:**

The online version contains supplementary material available at 10.1007/s10682-022-10209-1.

## Introduction

Climate change and habitat loss can impose stress on biological organisms, either directly (e.g., increased heat exposure due to higher temperatures), over short time periods (e.g., increases in oxidative stress leading to higher DNA damage), or over long time periods (e.g., increased mutation rates resulting from lower effective population sizes due to shrinking habitats). Furthermore, stressors can interact synergistically, potentially resulting in larger effects than individual stressors alone. Inferring the effects of stress on fitness requires a broad array of assays, ranging from molecular assays, to behavioral studies, to an examination of species range shifts (e.g., the heat shock response [HSR] (Lindquist 1986); diel vertical migrations (e.g., Müller et al. 2018); shifts in species distributions towards higher elevations (e.g., Freitas et al. 2016; reviewed in Parmesan 2006; Pinsky et al. 2020; Walther et al. 2002)). Changes in gene expression, however, represent an early, immediate, measurable response that can be assayed in a laboratory environment under controlled conditions, and as such can be a useful indicator of the initial, direct impact of one or multiple stressors on large scale, long-term outcomes like fitness and distribution.

Here, we assay the changes in gene expression of two heat shock protein (HSP) genes (HSP90 and HSP60) in *Daphnia magna* in response to two different stressors—heat and mutation load. Members of the HSP gene family perform an array of essential functions in the cell, including acting as molecular chaperones, facilitating immune response, regulating apoptosis, and signaling protein degradation (Czarnecka et al. 2006; Höhfeld et al. 2001; Javid et al. 2007; Queitsch et al. 2002). HSP90 is a 90 kDa chaperonin, known as a ‘central modulator’ or a ‘hub of hubs’ due to its role in signaling pathways and protein–protein interactions (Schopf et al., 2017; Zabinsky et al., 2019b), that stabilizes a large clientele of intracellular proteins and signaling proteins. HSP60 is a 60 kDa chaperonin primarily localized to the mitochondria (Cheng et al. 1989), where it is involved in the de novo folding and refolding of imported proteins (Martin et al. 1992).

As molecular chaperones, HSPs stabilize proteins, protein complexes, and other molecular interactions, including under stressful conditions. Increased HSP expression has been observed, not only in response to heat, but also to a variety of other stressors (e.g., heavy metals, oxidative stress, cytotoxic agents, and mutation; Casanueva et al. 2012; Chen et al. 2018; Kim et al. 2014; Liu et al. 2015; Neuhaus-Steinmetz and Rensing 1997; Queitsch et al. 2002; Zabinsky et al. 2019a). In the case of mutation, specifically, HSP90 has been posited to act as a “capacitor of evolution” because it can mask mutations that cause protein misfolding or destabilization, thereby allowing cryptic variation to accumulate in populations (Rutherford and Lindquist 1998; Queitsch et al. 2002; Jarosz and Lindquist 2010). Conversely, when the cellular pool of HSP90 becomes depleted, this variation can be ‘released’, facilitating rapid evolution (Hummel et al. 2017; Jarosz et al. 2010; Mason et al. 2018; Queitsch et al. 2002; Rohner et al. 2013; Zabinsky et al. 2019a; Zhang et al. 2012). Evidence in support of this role for HSP90 includes higher mutation accumulation in HSP90 client proteins compared to non-client homologs (Lachowiec et al. 2015; Zabinsky et al. 2019b) and elevated HSP90 expression in hypermutator lines (Zabinsky et al. 2019a). Although fewer studies have looked at the capacity of HSP60 to play this role, there is evidence that upregulation of the bacterial homolog to HSP60, GroEL, can buffer mutations in a similar capacity to HSP90 (Sabater-Muñoz et al. 2015).

Although most studies of HSP responses have examined responses to stressors individually, as individuals increasingly contend with combinations of stressors, understanding their interactions will become more important (Côté et al. 2016; Jackson et al. 2021). In fact, the effects of multiple stressors often depart from a simple additive model—for example, in *Arabidopsis*, 61% of genes responsive to a dual stress did not respond to either of the two stresses alone (Rasmussen et al. 2013) and there are syngeristic effects of stress on life-history traits in *Daphnia* in cases where multiple stressors are applied (Cuenca‐Cambronero et al. 2021). Furthermore, heat shock and caloric restriction have been found to have a synergistic effect on HSP expression in *C. elegans* (Raynes et al. 2012), and heat shock and treatment with a variety of pharmaceutical agents have been found to synergistically increase the levels of HSP expression in in a large variety of insects, aquatic organisms, and in cell culture (Mahmood et al. 2014; Török et al., 2003; Westerheide et al. 2004). Here, we quantify HSP90 and HSP60 expression changes in response to heat shock and mutation using 5 genotypes of *Daphnia magna* from across a latitudinal gradient. We predicted both mutation and heat shock will be associated with an increase in HSP expression, but did not anticipate the degree to which stressors might have synergistic effects or how different the influence of stress would be on each of the loci. We did not predict an effect of genotype, but could imagine genotypes collected from along a latitudinal gradient would have distinctive expression profiles. *Daphnia* (Cladocera) have served as an ecological, evolutionary, and ecotoxicological model for well over a century (Schaack 2008; Shaw et al. 2008; Yampolsky et al. 2014), in part due to their cosmopolitan distribution, however  genomic resources are now available as well (Colbourne et al. 2011; Lee et al. 2019; Orsini et al. 2016). Previously, the *Daphnia* system has been used to demonstrate differences in gene expression, protein production, and microevolutionary change at HSP loci in the lab in response to environmental change (Becker et al. 2018; Mikulski et al. 2009, 2011; Pauwels et al. 2007).

## Materials and methods

### Experimental design and study system

**Fig. 1:**
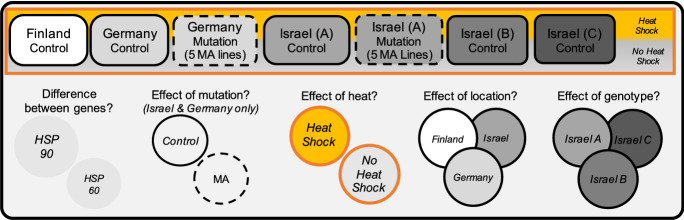
Experimental design showing all 15 genotypes assayed (top) in triplicate to quantify HSP60 and HSP90 expression levels to answer five questions (bottom). Genotypes were from Finland (n = 1), Germany (n = 1), and Israel (n = 3, **A**–**C**; solid border). In addition, some genotypes assayed were from mutation accumulation lines (n = 10; dashed borders) derived from genotypes collected in Germany and Israel. Assays were performed on individuals raised in a common laboratory environment and exposed to one of two environmental conditions (no heat shock [gray] or heat shock [yellow])

Our experimental design allowed us to assess the impact of heat stress and mutation accumulation on gene expression for two members of the HSP gene family (HSP60 and HSP90) in multiple genotypes originating from multiple locations in the aquatic microcrustacean, *Daphnia magna* (Order: Cladocera), which use a facultative parthenogenetic reproductive strategy (Fig. 1).

The individual animals used in this study were derived from genotypes originally collected in Finland, Germany, and Israel (provided by D. Ebert in 2014). These three locations form a latitudinal gradient and experience distinctive environmental regimes (including temperatures, periods of dry down, and census population sizes; Lange et al. 2015; see Supplemental Table S1a). We assayed one genotype from Finland (FC), one genotype from Germany (GA), and three different genotypes from a single population in Israel (IA, IB, and IC; Fig. 1). For the genotype from Germany (GC) and one of the genotypes from Israel (IA), both descendants of the originally collected genotypes (referred to as ‘control lines’ hereon) and descendants of five mutation accumulation (MA) lines were assayed (average number of generations of MA = 24; Table S1b). In brief, mutation accumulation experiments date back to at least the 1950s (e.g., Paxman 1959) and, generally, involve initiating lines and propagating each line separately via single progeny descent. The non-competitive environment and random selection of the individuals used to propagate the line each generation allows any mutations that occur to be passed down (‘accumulated’), and the descendent individuals can be compared against descendants of parallel lineages where mutations are not accumulated because selection was not minimized by removing competition for resources. The complete details for the MA experiment performed to generate these lines are described in Ho et al. (2019).

All control and MA lines were arranged haphazardly to randomize their position in the rack and reared in a common laboratory environment (at 18 °C) prior to this experiment, and the individuals used in the assay were from fourth generation descendants to minimize any maternal effects. Although we set up 4 biological replicates for each lineage/condition combination for the heat shock exposure (described below), in some cases individuals did not survive until the end of the experimental period. In most cases, enough individuals survived such that we were able to perform RNA extractions and downstream molecular analyses on 2–3 biological replicates for each lineage/condition assayed (n = 168 total) which then were assayed using quantitative PCR (see below) using three technical replicates (see Table S1c for sample lists). All individuals in the experiment were reared in pairs in 40 mL of Aechener Daphnien Medium (ADaM; Klüttgen et al. 1994) in 50 mL plastic conical tubes in environmental chambers during a 15 day period in June/July 2019 with 16:8 light:dark cycle at constant temperature and humidity.

### Heat shock exposure

After 15 days of growth and regular feeding, individual animals were transferred to 1.7 mL microcentrifuge tubes in 500 µL of ADaM to perform an acute heat shock. From each pair of individuals, one was placed in a 30 °C Corning LSE Digital Dry Bath inside of an 18 °C Percival incubator (heat shock), and the other half were placed in a Corning LSE Digital Dry Bath that was turned off and equilibrated to ambient temperature inside of the same 18 °C Percival incubator (no heat shock control). 30 °C has been used in the past as an acute heat shock condition to measure transcriptomic response to stress in *Daphnia* (Becker et al. 2018). Individuals were heat shocked for 2 h. After 2 h, the ADaM was removed and replaced with 300 µL 1X DNA/RNA Shield from the Zymo Research Quick-RNA Miniprep Kit. Samples were frozen immediately in liquid nitrogen and stored at − 20 °C.

### RNA extraction and reverse transcription

RNA was extracted from each sample independently using the Zymo Research Quick-RNA Miniprep kit according to the manufacturer’s protocol. Briefly, one *D. magna* individual in 1X DNA/RNA Shield was mixed with 300 µL RNA Lysis Buffer and ground using a microcentrifuge pestle. All centrifugations were done at 10,000*g* for 30 s unless specified with a Labnet Spectrafuge 24D. After centrifugation through a DNA-specific filter for 1 min, the flow-through was mixed with 600 µL ethanol, transferred to an RNA-specific filter, and centrifuged. The bound RNA was then washed with 400 µL of RNA Wash Buffer and treated with a solution of 75 µL DNA Digestion Buffer and 5 µL DNase I for 15 min in order to destroy any remaining DNA. The digestion was centrifuged, and the remaining RNA was washed once with 400 µL RNA Prep Buffer and once with 700 µL RNA Wash Buffer. The final wash was done with 400 µL RNA Wash Buffer, and it was centrifuged for 2 min in order to remove any latent buffer. RNA was then eluted into a nuclease-free microcentrifuge tube with 50 µL DNase/RNase-free water and stored at − 20 °C. Concentration of RNA was measured using the Invitrogen Qubit RNA BR Assay with a Qubit 3.0 (Life Technologies). For each sample, 100 ng of total RNA per individual was reverse transcribed with random primers in a 20 µL reaction using the Promega GoTaq 2-Step RT-qPCR System according to the manufacturer’s protocol. cDNA was then stored at − 20 °C.

### Quantitative PCR

An RNA sequence for HSP60 was obtained from Steinberg et al. (2010) and the sequence for HSP90 from Kotov et al. (2006). Sequences were aligned to whole genome sequences of control lines from each genotype in this study using blastn (see Supplemental Data File A for alignments). Candidate control genes for qPCR (succinate dehydrogenase (SDH), glyceraldehyde-3-phosphate dehydrogenase (GAPDH), and ubiquitin conjugating protein (UBC)) were selected from Heckmann et al. (2006). Primers were designed using Primer3 to generate amplicons between 70 and 200 bp (Supplemental Table S0). After qPCR, the stability of each control gene was checked using RefFinder (Xie et al. 2012). Though UBC expression was previously observed to be somewhat responsive to heat in different *D. magna* populations (Jansen et al. 2017), we found it to be the most stable, so it was used as the reference gene for normalizing HSP60 and HSP90 expression. Primer efficiencies were assessed by serial dilution. Both target genes and UBC were found to have efficiencies of 100% (Supplemental Figure 1). Any primer pairs with estimated efficiencies slightly over 100% were assumed to have true efficiencies of 100%. Primer functionality and specificity were verified through end-point PCR using Qiagen Taq PCR Master Mix. Products were analyzed by gel electrophoresis. Amplicon lengths are as follows: HSP90 is 138 bp, HSP60 is 74 bp, and UBC is 90 bp.

We performed qPCR using the Promega GoTaq 2-Step RT-qPCR System according to the manufacturer’s protocol. Each 10 µL reaction included 5 µL GoTaq qPCR Master Mix, 2 µL each of 1 µM forward and reverse primers, and 1 µL of cDNA. Cycling conditions (CFX Connect, Bio-Rad) were 2 min at 95 °C for polymerase activation followed by 40 cycles of 15 s of denaturation at 95 °C with 1 min at 55 °C of annealing and extension. Lastly, a melt curve from 55 to 95 °C was added at the end to verify no off-target amplification. Samples and genes were organized through the sample maximization method such that each plate only amplified one gene, but each plate had all samples (2–3 biological replicates per line and treatment). Three technical replicate reactions were performed on separate plates. Because each sample was represented in every plate, plates served as technical replicates (Derveaux et al. 2010).

### Data analysis

In order to determine if any technical replicates were outliers, the mean of each sample x gene combination was calculated. Only replicates < 1 standard deviation from the mean (− 1 < Z-score < 1) were included in the analysis. The relative quantity (RQ) of experimental genes (HSP90, HSP60) originally present in the sample was calculated using the mean quantification cycle (C_q_) of the remaining replicates, as determined by the CFX software, and the efficiency of the primer pair (E). Experimental gene RQs were normalized by the RQ of the reference gene (UBC) as described by Rieu and Powers (2009) to estimate normalized relative quantities (NRQ). NRQ values were log transformed prior to statistical analysis to correct for heterogeneity of variance (Rieu and Powers 2009). Data used for analyses can be found in Tables S6 and Table S7 for HSP90 and HSP60, respectively. Transformed data (using a log_2_(NRQ) transformation) are in Table S8 and Table S9, for HSP90 and HSP60, respectively. Raw data can be found in Table S10a, and data without outliers removed by Z-score can be found in Table S10b.

We tested our log-transformed dataset for normality and homogeneity of variances. Using the Levene’s test, the data for HSP90 (F_13,70_ = 1.56, *p* = 0.117) and HSP60 (F_13,70_ = 1.1, *p* = 0.38) suggest that there is homogeneity of variances. Through a Shapiro-Wilks test on the residuals of a multiple linear regression model including all data for both genes independently, HSP60 did not depart significantly from normality (W = 0.974, *p* = 0.087) while HSP90 expression levels were found to have high non-normality (W = 0.811, *p* < 0.0001). As the data were already log_2_ transformed, there was no further transformation that improved the normality of the dataset. However, because there is no non-parametric equivalent of a multi-way ANOVA, and ANOVA is robust to departures from normality (Knief and Forstmeier, 2020) such as those in this dataset, differences in means were tested using ANOVAs.

All ANOVAs were performed in R using the ‘aov’ function with its default formula parameters (R Core Team 2017); see Supplemental File S1 for all R code). The full 5-Way ANOVA model tested the effects of gene (HSP60, HSP90), heat shock, mutation accumulation, location of origin, and genotype, and all interactions, on expression level of both HSP60 and HSP90 (Model A in R code and Table S2). To test for mutation accumulation effects specific to HSP90 and HSP60, a model was made for each gene with all samples including both mutation accumulation lines and control lines for all genotypes using a 4-Way ANOVA (Models B and C, respectively in R code and Tables S3 and S4). To test for location-of-origin effects, in addition to Model A, two additional models (Model D and E) were made that included only control lines using a 2-Way ANOVA (Tables S3 and S4). Lastly, two 2-Way ANOVA models were made using only Israel control lines for each gene to test for a genotype effect within a single location (Models F and G in R code and Table S5).

## Results

**Fig. 2:**
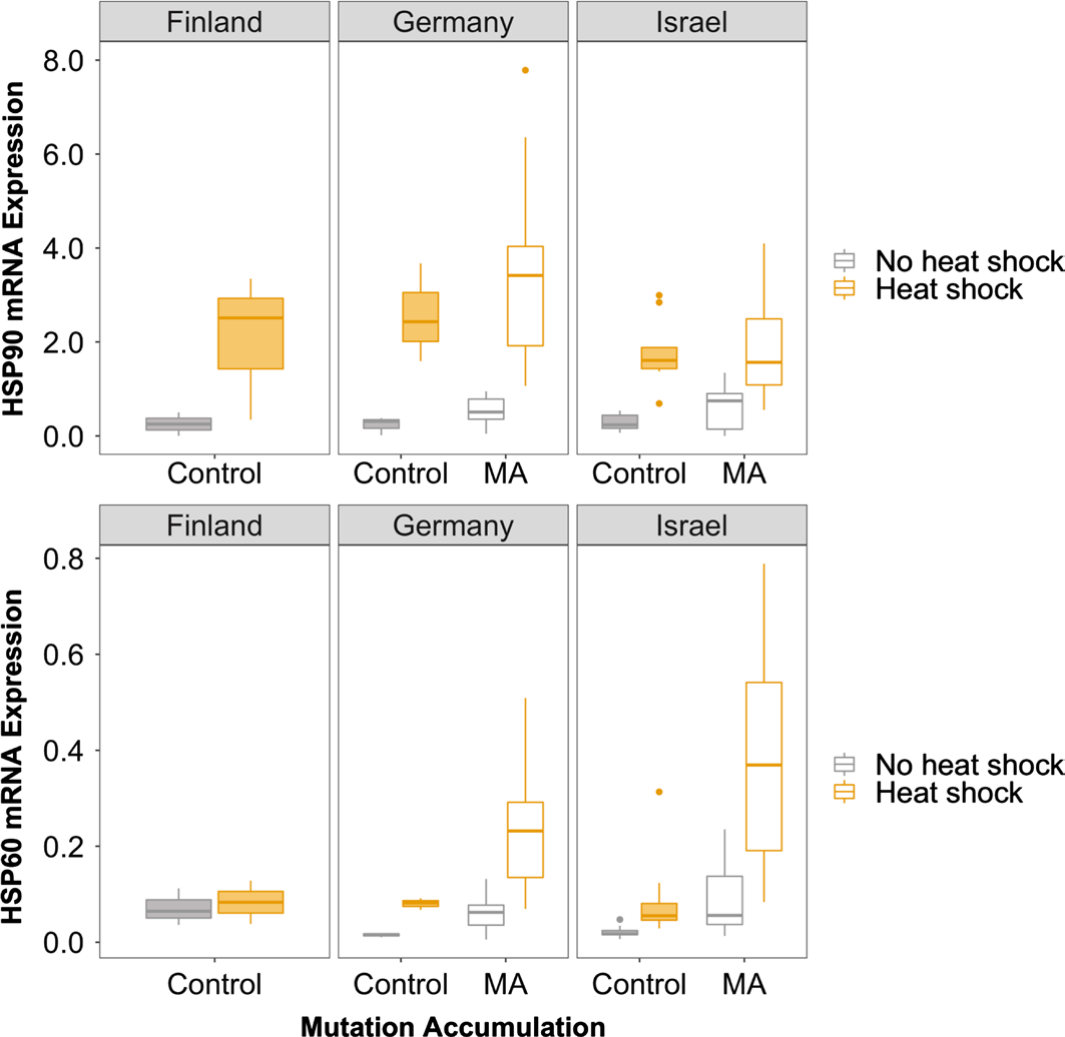
Gene expression for HSP90 (top) and HSP60 (bottom) in genotypes collected originally from three locations (Finland, Germany, and Israel) from individuals from mutation accumulation (unshaded) versus control lines (shaded) that were (yellow) and were not heat shocked (gray). Horizontal lines represent medians, boxes indicate quartiles and vertical lines illustrate the maximum value of 1.5× IQR + the 75th percentile and the minimum value of the 25th percentile—1.5× IQR of the variance. Note: One outlier in Germany MA (HSP90 mRNA Expression = 12.64) was excluded from the graph of HSP90 expression to better visualize differences in medians; however, it is included in the ANOVA results in Table 1

Our assay of gene expression levels for HSP60 and HSP90 allowed us to assess the effects heat stress (30 °C vs. 18 °C) and mutation loads across genotypes of *D. magna*. Overall, HSP90 was expressed approximately tenfold higher than HSP60 (F = 163.7, *df* = 1, *p* << 0.001; Tables 1 and 2; Table S2). Generally, heat shock increases the mean expression levels of both genes ~ 5.9× (F = 101.2, *df* = 1, *p* < 0.001; Table 1), although the specific fold-change depends on the gene and location-of-origin (Table 2 and Fig. 2). We also observed higher HSP expression levels in lineages with higher mutation loads (MA lines relative to control lines; F = 15.7, *df* = 1, *p* < 0.0001; Table 1 and Fig. 2), although the size of the increase was not as large as with heat shock (on average, 3.8×; Table 2) and was only significant for HSP60 (F = 42.9, *df* = 1, *p* < 0.0001; Table 1 and Tables S3 and S4).

**Table 1:** Analysis of variance (ANOVA) for gene expression based on transcript abundance for HSP60 and HSP90 assayed in *Daphnia magna* originally collected from three locations (Finland, Germany, and Israel), subject to mutation accumulation, and raised with and without heat shock

Data partitions	Factor	*Df*	Sum of squares	F value	Pr(> F)
All data					
Main effects and 2/3-way interactions	Location	2	6.63	1.2611	0.2865
	**Gene**	1	430.19	163.7196	**< 0.0001**
	**HeatShock**	1	265.8	101.1594	**< 0.0001**
	**MutationAccumulation**	1	41.23	15.6915	**0.0001**
	Location:Gene	2	10.44	1.9864	0.1410
	Location:HeatShock	2	3.68	0.701	0.4978
	Gene:HeatShock	1	7.62	2.8984	0.0909
	Location:MutationAccumulation	1	1.73	0.659	0.4183
	**Gene:MutationAccumulation**	1	15.4	5.861	**0.0168**
	HeatShock:MutationAccumulation	1	1.37	0.5202	0.4720
	Location:Genotype	2	7.37	1.4027	0.2494
	**Location:Gene:HeatShock**	2	19.91	3.7878	**0.0250**
HSP 60 only					
Main effects	Location	2	0.819	0.3281	0.7214
	**HeatShock**	1	92.799	74.3486	**< 0.0001**
	**MutationAccumulation**	1	53.519	42.8784	**< 0.0001**
HSP 90 only					
Main effects	Location	2	13.637	1.7017	0.1898
	**HeatShock**	1	183.224	45.726	**< 0.0001**
	MutationAccumulation	1	3.118	0.7782	0.3807
HSP 60 only, Israel only					
Genotype effects	Genotype	2	4.0676	3.0971	0.0823
	**HeatShock**	1	16.6449	25.35	**0.0003**
	Genotype:HeatShock	2	3.9055	2.97	0.0893
HSP 90 only, Israel only					
Genotype effects	**Genotype**	2	5.729	6.3804	**0.0130**
	**HeatShock**	1	34.261	76.3196	**< 0.0001**
	Genotype:HeatShock	2	1.048	1.1673	0.3442

Factors with statistical significance based on an alpha value of 0.05 are in bold

For complete ANOVA tables of all data partitions, see Supplemental Tables S2–S5; for the raw data used in this analysis, see Supplemental Tables S6, S7, and S10

**Table 2:** Estimated mean expression levels for HSP60 and HSP90 assayed in *Daphnia magna* originally collected from three locations (Finland, Germany, and Israel), subject to mutation accumulation, and raised with and without heat shock

Gene	Location	Genotype	Mutation accumulation	Heat shock	Mean expression (NRQ) untransformed
HSP90	Finland	FC	No	−	0.350
		FC	No	+	2.282
	Germany	GC	No	−	0.181
		GC	No	+	2.754
		GC	Yes	−	0.674
		GC	Yes	+	4.267
	Israel	IA, IB, IC	No	−	0.314
		IA, IB, IC	No	+	1.791
		IA	Yes	−	0.636
		IA	Yes	+	1.818
		IA	No	−	0.124
		IA	No	+	1.287
		IB	No	−	0.392
		IB	No	+	1.917
		IC	No	−	0.425
		IC	No	+	2.169
HSP60	Finland	FC	No	−	0.071
		FC	No	+	0.083
	Germany	GC	No	−	0.015
		GC	No	+	0.080
		GC	Yes	−	0.059
		GC	Yes	+	0.245
	Israel	IA, IB, IC	No	−	0.021
		IA, IB, IC	No	+	0.090
		IA	Yes	−	0.090
		IA	Yes	+	0.384
		IA	No	−	0.010
		IA	No	+	0.135
		IB	No	−	0.019
		IB	No	+	0.042
		IC	No	−	0.035
		IC	No	+	0.094

For Germany and Finland, one genotype each was sampled (GC and FC, respectively). For Israel, three individual genotypes were assayed (IA, IB, and IC). For complete ANOVA tables of all data partitions, see Supplemental Tables S2–S5; for the data used in this analysis, see Supplemental Tables S6, S7, and S10.

**Fig. 3:**
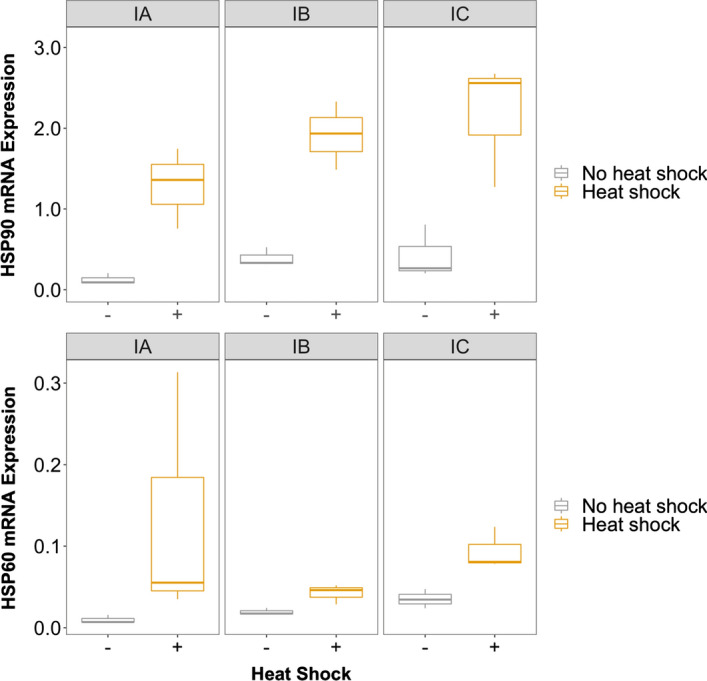
Gene expression levels for HSP90 (top) and HSP60 (bottom) with exposure to heat shock (yellow) and without heat shock (gray) for three genotypes from Israel (data for ANOVAs appears in Table S5). Horizontal lines represent medians, boxes indicate quartiles and vertical lines illustrate the maximum value of 1.5× IQR + the 75th percentile and the minimum value of the 25th percentile—1.5× IQR of the variance

In lineages where both heat stress occurs and high mutation loads have accumulated, the change in HSP expression observed is higher than a purely additive model would predict (a ~ 23× increase compared to a ~ 9.8× null expectation, but there is no significant interaction effect likely due to the (expected) increase in variance introduced by heat and mutation accumulation, as well [Fig. 2 and Table 2; Table S2]). Even though genotypes were originally sampled from a latitudinal gradient from Finland to Israel, there is little evidence of intraspecific variation in HSP expression overall (no location-of-origin main effect [F = 1.26, *df* = 2, *p* = 0.29]; Table 1), although there was an effect of genotype for one locus (meaning genotypes IA, IB, and IC from Israel exhibited high variation for HSP 90 [F = 6.4, *df* = 2, *p* = 0.01], but not HSP60 [F = 3.1 *df* = 2, *p* = 0.08; Table S5]; Fig. 3).

## Discussion

The HSP genes are members of a large and diverse family and play a variety of important roles in responding to extrinsic and intrinsic sources of cellular stress and molecular destabilization, including heat and mutation (Kim et al. 2014; Liu et al. 2015; Neuhaus-Steinmetz and Rensing 1997). While HSP90 has long been referred to as a mutational “capacitor” because of its major role in protein folding and the large number of proteins it interacts with (Schopf et al. 2017), the role of HSP60 in the stress response is less well understood given its localization primarily to the mitochondria (Magnoni et al. 2014). Given recent studies reporting the highest direct estimates of spontaneous mutation rates in *D. magna* (based on mutation accumulation experiments with animals; Ho et al. 2019, 2020, 2021; Ho and Schaack 2021), in addition to their long-standing importance as an ecological and environmental model system, understanding of the stress response of *Daphnia* and their ability to buffer the phenotypic effects of mutation is of particular interest (Davenport et al. 2021; Latta et al. 2015).

Here, we compare the expression levels of HSP90 and HSP60 with and without heat stress in MA lines versus controls lines where mutations did not accumulate across individuals from five different genotypes. Overall, we find that HSP90 is expressed ~ 10× more than HSP60 in *D. magna* (Table 1 and Fig. 2). This corroborates previous work that shows HSP90 constitutes approximately 1–2% of the total protein content of eukaryotic cells (Borkovich et al. 1989). The importance of this protein is underscored by its abundance and its interaction with other proteins (e.g., in yeast, HSP90 interacts with up to 20% of other proteins produced in the cell; Taipale et al. 2010). As predicted, we found both genes have a robust heat shock response (Table 1 and Fig. 2), likely because elevated HSP expression, generally, can protect against exposure of hydrophobic segments, aggregation, and misfolding of destabilized proteins (Kimura et al. 1993; Vabulas et al. 2010; Chen et al. 2021). In *D. melanogaster*, HSP60 is upregulated in response to heat (Martin et al., 1992) and oxidative stress (Singh et al. 2009), but a rapid response may be even more important in aquatic animals living in shallow water given the major temperature fluctuations they experience (Feder and Hofmann 1999).

We also observed an increase in HSP expression in mutation accumulation lines relative to controls, especially in HSP60 (Table 1). These elevated levels of HSP60 may be related to the higher mutation rates observed in the mtDNA genome relative to the nuclear genome, prevalent in animals (reviewed in Schaack et al. 2020) and observed in *D. pulex* (Xu et al. 2012) and *D. magna* (Ho et al. 2020). Mutations can lead to toxic protein misfolding and aggregation (Bross et al. 1999). Molecular chaperones, including HSPs, recognize misfolded proteins and facilitate their removal and, therefore, proteome maintenance (proteostasis; Guo et al. 2019; Samant et al. 2018). HSP60 specifically is involved in mitochondrial proteostasis and is upregulated in response to several cancers (Guo et al. 2019), suggesting that it responds transcriptionally to mutation. The greater upregulation of HSP60 in response to mutation accumulation underscores the importance of further examining the potential of other HSPs (in addition to HSP90) as potential mutational capacitors (Rutherford and Lindquist 1998; Bernatowicz et al. 2021).

Notably, the application of both stressors simultaneously (heat shock and mutation loads) led to an increase in gene expression levels at both loci beyond the levels predicted by purely additive effects, suggesting the synergism between multiple stressors as a result of global climate change and habitat loss could have compounding effects. While examining gene expression changes when stressors are applied potentially represents and early and immediate response, there is reason to think that HSPs in particular represent a relatively complex protein/phenotype given that they play so many roles in the cell. Finally, because the HSP expression variance was greater in cases where two stressors were applied (Fig. 2), it is possible that evolutionary forces shaping the traits affected by HSP expression will have a greater range of trait values on which to act, should these stressors become more prevalent in natural environments.

Initially, we were surprised by the lack of difference in baseline HSP expression among genotypes originating from different locations (Table 1), given the abiotic differences among those locales (e.g., mean annual temperatures are ~ 2, 10, and 21 °C in Finland, Germany, and Israel, respectively; Rohde and Hausfather 2020). It could be that evolution of the HSR depends more on maximum temperatures or temperature fluctuations, however, which exhibit a much smaller range of only ~ 10 and 7 degrees, respectively, among these populations (Table S1a; Cambronero et al. 2018; Gehring and Wehner 1995; Hofmann and Somero 1996; Sgrò et al. 2010; Tomanek 2010). The lack of intraspecific variation in HSP expression observed is consistent with protein level studies (Bernatowicz et al. 2021), and may be explained by the high degree of sequence identity at these two loci (> 99% of sites are identical for coding regions in HSP60 [418/422] and HSP90 [735/741]; Supplemental Data Files).

In conclusion, our results provide support for HSPs playing a role in both responding to higher temperatures and dealing with intrinsic sources of intracellular stress, like mutation. These dual roles may be important, especially for *Daphnia*, in a changing climate where higher mean temperatures, larger temperature fluctuations, and habitat loss might increase heat exposure and/or lead to elevated mutation rates. Indeed, while a changing climate can alter exposure to UV or other atmospheric mutagens, directly, it can also reduce the availability of freshwater aquatic habitats caused by drought or sea level rise, thereby reducing effective population size, and thus reducing the relative role of selection in shaping the evolution of the mutation rate, as predicted by the Drift Barrier Hypothesis (reviewed in Lynch et al. 2016). More directly, heat stress itself has been shown to result in higher mutation rates, which might provide an additional role for HSPs in the buffering of mutations (Chu et al. 2018). While spontaneous mutations are known to be, on average, deleterious, beneficial mutations do occur and can provide an evolutionary escape hatch or opportunity for rapid adaptation (Swings et al. 2017; Rutter et al. 2018; Weng et al. 2021). Ultimately, genetic variation generated by mutations, if it can be buffered against in the short term, may facilitate the long-term success of organisms as climates change.

## Supplementary Information

Below is the link to the electronic supplementary material.

Supplementary file 1 (TXT 26 kb)

Supplementary file 2 (ZIP 6 kb)

**Table S0.** Primers used for RT-qPCR with *Daphnia magna* cDNA extracts in this study. **Table S1a, b, and c.** Sample collection data for *Daphnia magna* and sample sizes for in this study. **Table S2a and b.** Complete multifactor ANOVA table conducted using the entire dataset of expression level data for *Daphnia magna* across genotypes, heat shock treatments, with and without mutation accumulation, including main and interaction effects and an ANOVA table using *only* data for lineages where both heat and mutation accumulation effects can be tested (genotypes IA and GC). **Table S3.** Partitioned ANOVAs performed using HSP90 expression levels based on log2(NRQ) data found in Table S8. **Table S4.** Partitioned ANOVAs performed using HSP60 expression levels based on log2(NRQ) data found in Table S9. **Table S5.** Partitioned ANOVAs performed using HSP90 and HSP60 expression levels based on log2(NRQ) data for only Israel lines to test for genotype effects. **Table S6.** HSP90 expression levels in *Daphnia magna* before log transformation (used in Fig. 2 and Fig. 3). **Table S7.** HSP60 expression levels in *Daphnia magna* before log transformation (used in Fig. 2 and Fig. 3). **Table S8.** Calculations of the log2(NRQ) for HSP90 across all available genotypes and biological replicates used for all statistics unless otherwise specified. **Table S9.** Calculations of the log2(NRQ) for HSP60 across all available genotypes and biological replicates used for all statistics unless otherwise specified. (XLSX 163 kb)

**Supplemental Figure 1**. Amplification Curves of Dilution Series for qPCR Primers. Each standard curve was made by using the standard qPCR reaction mix and thermocycler program with two replicates of a dilution series of 1, 1/4, 1/16, and 1/64 of the original cDNA concentration. A) Standard amplification curve of HSP90 with an efficiency = 100.3%, B) standard amplification curve of HSP60 with efficiency = 102.6%, C) standard amplification curve of UBC with efficiency = 101.8%. (JPG 432 kb)

**Supplemental Figure 2**. Q–Q plots of HSP90 mRNA expression levels (A) and HSP60 mRNA expression levels (B). Q–Q plots were made from residuals of a multiple linear regression model using all samples for both genes independently (JPG 123 kb)

## Data Availability

All raw and transformed data used in this study are in Supplemental Tables S6, S7, S8, S9, and S10. All R code (Supplementary file 1) and sequence data (Supplementary file 2) have been uploaded as a Supplemental File.
