# Supplementary figures and images for: Thermal stress and mutation accumulation increase heat shock protein expression in Daphnia
Source: Evol Ecol. 2022 Sep 6;36(5):829–44. doi: 10.1007/s10682-022-10209-1 (PMC9522699; doi:10.1007/s10682-022-10209-1)

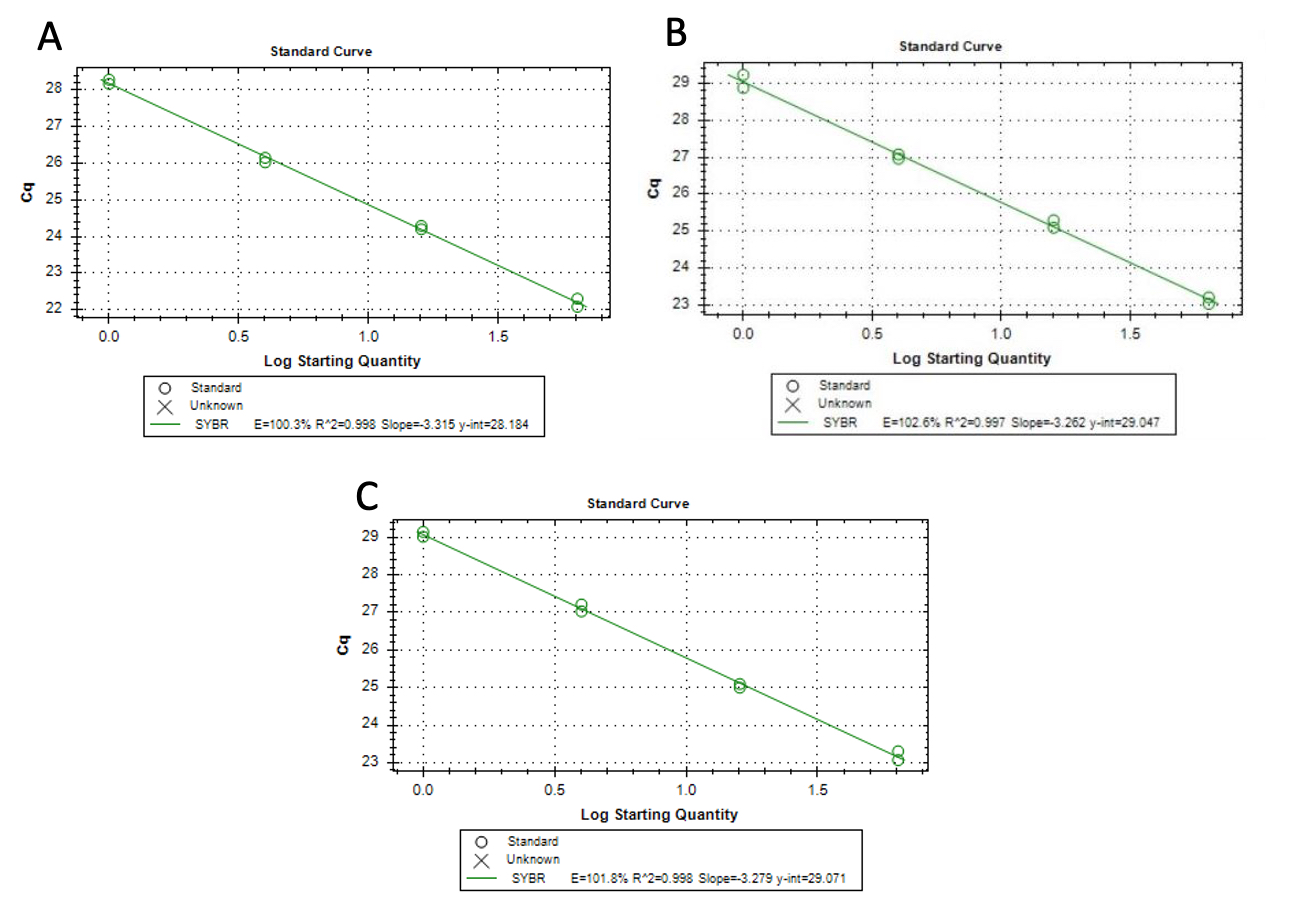

Supplement: Supplementary file 4 — Supplemental Figure 1. Amplification Curves of Dilution Series for qPCR Primers. Each standard curve was made by using the standard qPCR reaction mix and thermocycler program with two replicates of a dilution series of 1, 1/4, 1/16, and 1/64 of the original cDNA concentration. A) Standard amplification curve of HSP90 with an efficiency = 100.3%, B) standard amplification curve of HSP60 with efficiency = 102.6%, C) standard amplification curve of UBC with efficiency = 101.8%. (JPG 432 kb) [file 10682_2022_10209_MOESM4_ESM.jpg]

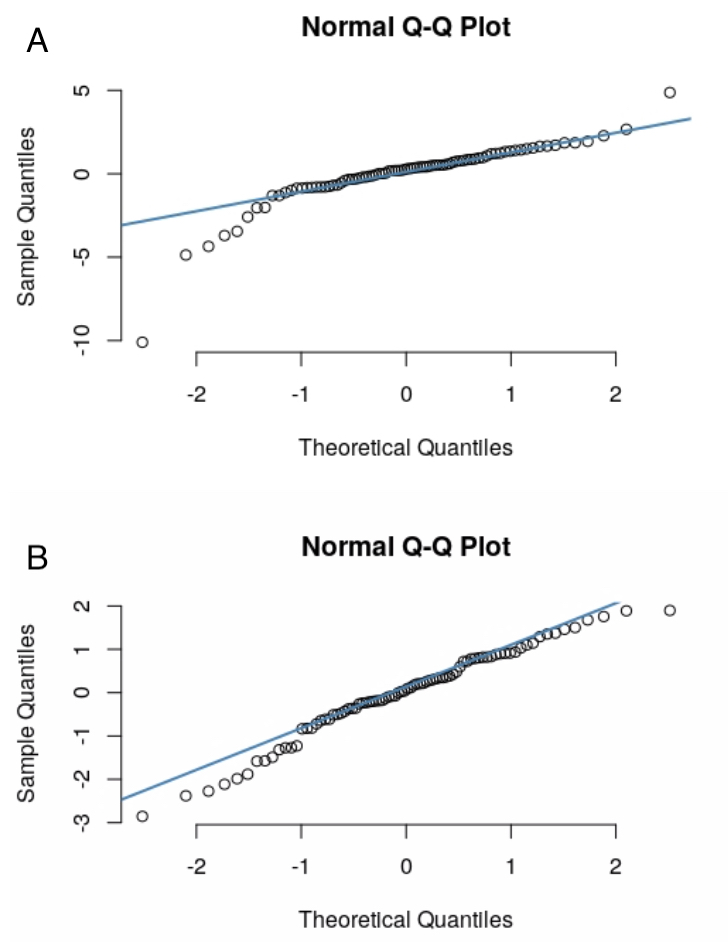

Supplement: Supplementary file 5 — Supplemental Figure 2. Q–Q plots of HSP90 mRNA expression levels (A) and HSP60 mRNA expression levels (B). Q–Q plots were made from residuals of a multiple linear regression model using all samples for both genes independently (JPG 123 kb) [file 10682_2022_10209_MOESM5_ESM.jpg]
